# The speed of parietal theta frequency drives visuospatial working memory capacity

**DOI:** 10.1371/journal.pbio.2005348

**Published:** 2018-03-14

**Authors:** Nina Wolinski, Nicholas R. Cooper, Paul Sauseng, Vincenzo Romei

**Affiliations:** 1 Centre for Brain Science, Department of Psychology, University of Essex, Colchester, United Kingdom; 2 Department Psychologie, Ludwig-Maximilians-Universität München, München, Germany; 3 Centro studi e ricerche in Neuroscienze Cognitive, Dipartimento di Psicologia, Università di Bologna, Campus di Cesena, Viale Europa, Cesena, Italy; University of Birmingham, United Kingdom of Great Britain and Northern Ireland

## Abstract

The speed of theta brain oscillatory activity is thought to play a key role in determining working memory (WM) capacity. Individual differences in the length of a theta cycle (ranging between 4 and 7 Hz) might determine how many gamma cycles (>30 Hz) can be nested into a theta wave. Gamma cycles are thought to represent single memory items; therefore, this interplay could determine individual memory capacity. We directly tested this hypothesis by means of parietal transcranial alternating current stimulation (tACS) set at slower (4 Hz) and faster (7 Hz) theta frequencies during a visuospatial WM paradigm. Accordingly, we found that 4-Hz tACS enhanced WM capacity, while 7-Hz tACS reduced WM capacity. Notably, these effects were found only for items presented to the hemifield contralateral to the stimulation site. This provides causal evidence for a frequency-dependent and spatially specific organization of WM storage, supporting the theta–gamma phase coupling theory of WM capacity.

## Author summary

Our ability to temporarily retain sensory information is limited to a handful of items and is referred to as working memory capacity. Such memory capacity has been shown to vary across the general population, with some people retaining a higher number of items than others. An influential theory suggests that this individual capacity might be determined by the speed of slow brain waves (so-called theta waves) that range in frequency between four and seven cycles per second. It is hypothesized that these theta waves act as glue for items to be memorised such that the slower the theta waves, the higher the number of items that can be clustered and retained in memory. We tested this hypothesis by applying to human participants noninvasive current stimulation at slower or faster theta frequencies over a part of the brain that is involved in visuospatial working memory during a visuospatial task. In line with this influential theory, we found that stimulation at slower theta frequencies enhanced working memory capacity relative to stimulation at faster theta frequencies, which instead reduced working memory capacity. These effects were limited to visual items processed by the stimulated brain areas, confirming the importance of theta waves for the organization of visuospatial working memory.

## Introduction

**Fig 1:**
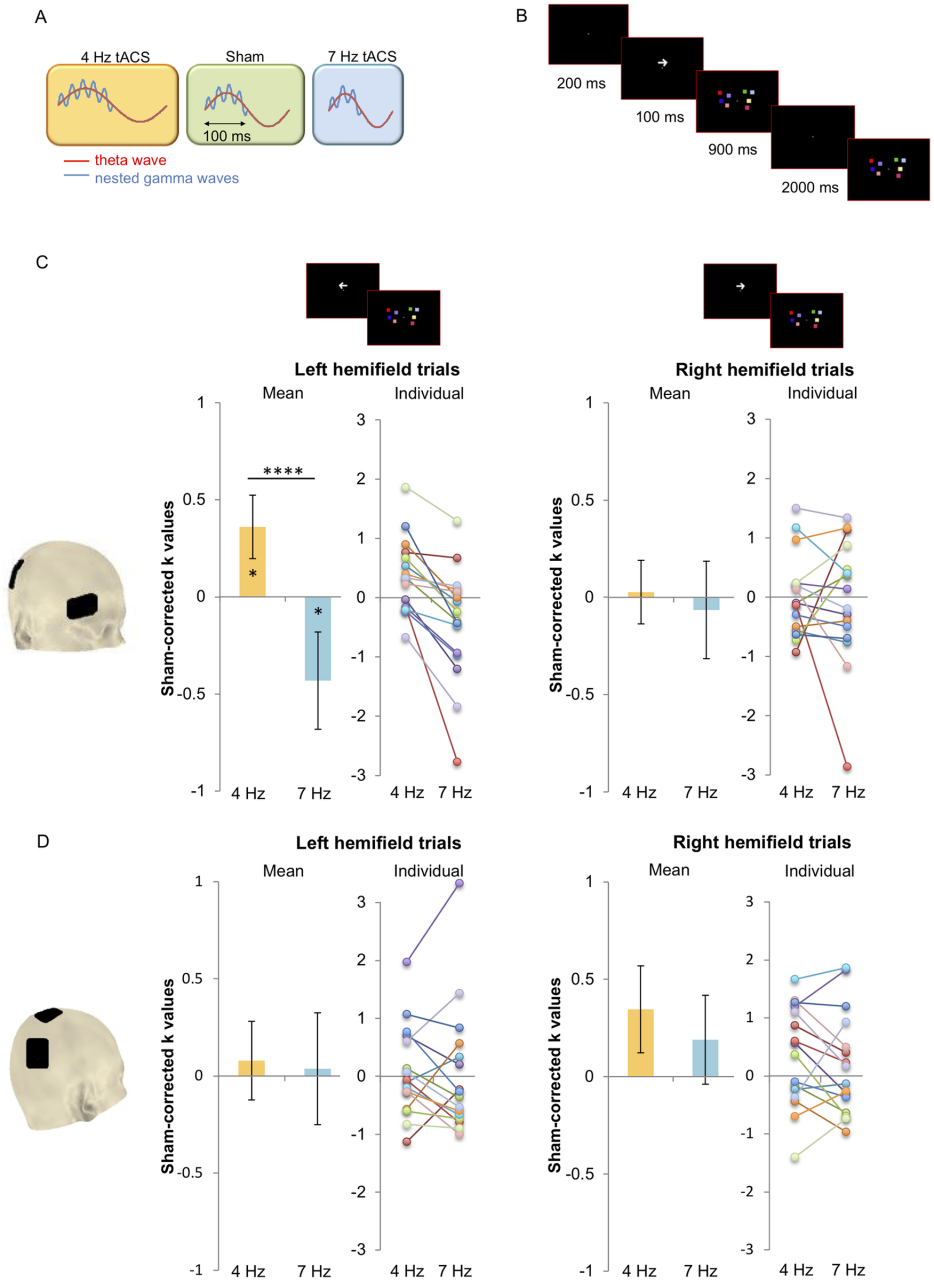
**(A) Theta–gamma phase coupling theory**. The maximum number of items stored in WM is thought to be a function of the number of gamma cycles nested into a theta wave [1,2]. We tested this theory by applying slower (4 Hz) and faster (7 Hz) theta frequency tACS, aiming at modulating the speed of theta cycles (as per the entrainment hypothesis; see [10,12,13,19]) to allow higher/lower numbers of gamma cycles nested within a theta phase. Four-hertz tACS (yellow panel) should slow down theta oscillations, allowing more gamma cycles to nest within a theta cycle, relative to sham (green panel), enhancing WM capacity. Seven-hertz tACS (blue panel) should speed up theta oscillations, allowing fewer gamma cycles to nest within a theta cycle, relative to sham (green panel), worsening WM capacity. **(B) Visual delayed match to sample task**. Two arrays of coloured squares were situated on either side of a white fixation cross in the centre of a black screen. The number of squares in each array (memory load) was 4, 5, or 6, with 20 trials presented for each load. The task started with a fixation cross on the screen. Prior to presentation of the arrays, an arrow appeared on the screen (200 ms) to indicate which of the two upcoming arrays (left or right) needed to be memorised. The two arrays then appeared on the screen (100 ms), followed by a retention interval (900 ms), again followed by two arrays (left and right; 2,000 ms). Participants had to indicate whether the array in the cued hemifield had changed. **(C) (Experimental Montage) and (D) (Control Montage): results** (underlying data can be found at: https://osf.io/rm6qp/). K-values for each combination of load and hemisphere for each condition per participant were calculated with the formula: (hit rate − false alarms) * set size [20] (see Data analysis). Leftmost graphs depict mean and individual K-values obtained for trials presented on the left hemifield for each active stimulation condition after sham correction (Sham-corrected 4 Hz, Sham-corrected 7 Hz). Rightmost graphs depict mean and individual K-values obtained for trials presented on the right hemifield for each active stimulation condition after sham correction (Sham-corrected 4 Hz, Sham-corrected 7 Hz). Significant differences between conditions were observed for the Experimental Montage (C) but not for the Control Montage (D) and only for stimuli presented to the left hemifield (i.e., contralateral to the stimulated parietal site). For non-sham-corrected K and accuracy data, see S1A and S1B Fig (S1A and S1B Table), respectively (underlying data can be found at: https://osf.io/rm6qp/). *p < 0.05; ****p <0.0001. Error bars depict standard error of the mean. tACS, transcranial alternating current stimulation; WM, working memory.

The theta–gamma cross-frequency coupling theory [1] (Fig 1A) proposes that individual fast brain waves (gamma cycles) represent individual memory items that are bound together to a multi-item memory by slow brain waves (theta oscillations). Consequently, individual differences in the length of a theta cycle (4–7 Hz) might determine how many gamma cycles (>30 Hz) can be nested into a theta cycle and may therefore determine memory capacity. This theory provides a potential neurophysiological mechanism for individual differences in the maximum number of items (number of gamma cycles) retained in the memory buffer (one theta cycle). According to this theory, it would be expected that slower theta frequencies will integrate a higher number of gamma cycles per theta cycle, resulting in increased memory capacity. Conversely, faster theta frequencies would bind a comparatively smaller number of nested gamma cycles, resulting in a decreased memory capacity.

Correlational studies have provided indirect support for this theory (e.g., [2,3]). For instance, Axmacher and colleagues [2] showed that increasing working memory (WM) load leads to a slowing down in the theta frequency. Moreover, recent neurostimulation work has shown that entraining parietal theta oscillations via transcranial alternating current stimulation (tACS) [4] or rhythmic Transcranial Magnetic Stimulation (TMS) [5] has proven effective in enhancing WM performance, providing causal evidence for the role of theta oscillations in WM performance. These works have so far mainly focused on enhancing theta amplitude by enhancing the theta signal-to-noise ratio, leading to better performance. A recent work has attempted to enhance WM capacity through manipulation of the intrinsic theta cycle length by frontal tACS set at a stimulation frequency slower than the individual theta [6]. However, it is unclear whether the enhanced WM performance obtained in that study is due to slowing of theta frequency or can be attributable to the more general impact of stimulation on theta amplitude, per se. Therefore, previous studies have left unanswered a long-lasting question regarding the exact mechanism by which theta oscillations orchestrate WM capacity: does the cycle length of theta oscillations play a mechanistic role in determining interindividual variability of WM capacity? Here, we test the prediction based on the theta–gamma phase coupling theory [1] that inducing slower theta cycles will enhance WM capacity, while inducing faster theta cycles will reduce WM capacity. We tested this prediction in a visuospatial WM paradigm based on seminal work by Vogel and Machizawa [7,8,9], who showed interindividual differences in visuospatial WM capacity to positively correlate with the amplitude of evoked responses localised over parietal areas contralateral to the hemifield where the stimulus to be kept in memory was presented. Crucially, using the same paradigm, Sauseng and colleagues [3] found a clear lateralisation of theta-locked gamma phase synchronization increase over parietal areas, again predicting individual WM capacity. Therefore, based on recent electroencephalography (EEG) [10], magnetoencephalography (MEG) [11], and behavioural evidence [12] that tACS can drive the intrinsic resonance frequency towards an externally imposed rhythm [13], we directly tested for the modulation of WM capacity by slow (4 Hz) and fast (7 Hz) theta tACS over parietal areas [3,7,8,9]. In line with our predictions, we found that slow theta tACS enhanced WM capacity while fast theta tACS reduced WM capacity. Importantly, these effects were specifically obtained for the visual hemifield contralateral to the stimulation site.

## Results

Two groups of 16 participants were each assigned to two different electrode montages. In both montages, an electrode was placed over the same right parietal region in order to stimulate the parietal area of the frontoparietal WM network, known to be relevant in visuospatial WM tasks [14]. This electrode was paired with either a return electrode over the vertex (Control Montage) or over the right supraorbital region (Experimental Montage). A few potential issues were anticipated with the use of the Control Montage for our paradigm to be effective: (a) the reduced distance between the two electrodes in the Control Montage, which might result in a significant proportion of current being shunted over the skin [15], rendering the stimulation less effective; (b) the spread of current across both hemispheres, due to the vertex electrode sitting centrally and therefore reducing the expected lateralised impact of stimulation on WM performance [3,7,8,9]; (c) the different orientation of current flow relative to the neurons’ orientation across the two montages, specifically due to the differential position of the return electrode, which may play a relevant role in the stimulation efficacy [16,17]; and (d) the Control Montage being less effective on the target brain area intraparietal sulcus (IPS) (see [18]). The Experimental Montage used was designed to overcome these potential confounds.

Participants in each montage group underwent active stimulation (at 4 and 7 Hz) and sham stimulation while performing a visuospatial delayed match to sample task [3,7,8,9]. The visuospatial WM task involved remembering an array of four to six coloured squares that was briefly presented to either the left or right visual hemifield (i.e., contralateral or ipsilateral to the stimulated hemisphere, respectively) for a short period of time and then assessing whether it was the same or different from a subsequently presented array (see Fig 1B for details on the task and stimuli example).

WM capacity across different memory loads was measured using a K-value, which is a standardised measure estimating how many items can be stored in WM (e.g., [3,7,8,9]). In addition, in order to make the results more comparable with other studies not using K as an estimate of memory capacity, the percentage of correct responses (accuracy) was also calculated. Finally, in order to reduce variability induced by the control condition sham (as the between-group factor), data were sham-normalised (for a non-sham-corrected data analysis, see S1 Fig).

A mixed factorial ANOVA with the between-factor Montage (Experimental Montage and Control Montage) and within-factors Condition (Sham-corrected 4 Hz, Sham-corrected 7 Hz) × Load (4, 5, and 6 items) × Hemifield (left and right) was carried out on the K-values (see Fig 1C and 1D, and Data analysis) and accuracy (see S1 Fig).

Results showed a main effect of Condition (K: F(1,30) = 5.90, *p* = 0.021, η^2^ = 0.16; accuracy: F(1,30) = 6.39, *p* = 0.017, η^2^ = 0.18), suggesting that stimulating at 4 Hz and 7 Hz relative to Sham had a differential impact on WM capacity. Importantly, a Condition × Hemifield × Montage interaction (K: F(2,60) = 5.79, *p* = 0.022, η^2^ = 0.16; accuracy: F(2,60) = 5.25, *p* = 0.029; η^2^ = 0.15) showed that the two montages modulated performance differently depending on stimulation Condition and Hemifield. Subsequent ANOVAs were therefore performed separately for each montage. In the Experimental Montage, we found a main effect of Condition (K: F(1,15) = 5.70, *p* = 0.03, η^2^ = 0.28; accuracy: F(1,15) = 5.75, *p* = 0.029, η^2^ = 0.28) and a significant interaction of Condition × Hemifield (K: F(1,15) = 9.53; *p* = 0.008; η^2^ = 0.39; accuracy: F(1,15) = 7.46; *p* = 0.015; η^2^ = 0.33), suggesting that the different stimulation conditions had a differential impact on left and right hemifields. Given the lateralised application of tACS (right parietal) and the contralateral parietal activation during visuospatial WM maintenance observed in previous work [3,7,8,9], a significant modulation of WM capacity was expected for items presented over the left (contralateral) but not the right (ipsilateral) hemifield. These trials were analysed separately in two further repeated measures ANOVAs (Condition × Load).

As expected, the analysis of the left hemifield trials showed a significant main effect of Condition (K: 4 Hz: 0.36 ± 0.016; 7 Hz: −0.43 ± 0.25; F(1,15) = 23.97; *p* = 0.0002; η^2^ = 0.61; accuracy: 4 Hz: 3.54% ± 1.71%; 7 Hz: −3.44% ± 1.94%; F(1,15) = 45.53, *p* = 0.000007, η^2^ = 0.75), while no main effect of Load nor interactions reached significance (K: all *p* > 0.42; accuracy: all *p* > 0.78). Moreover, one-sample *t* tests against 0 confirmed that 4-Hz tACS significantly enhanced K-values (and accuracy) relative to sham (K: *t*(15) = 2.28; *p* = 0.019, one-tailed; Cohen’s *d* = 0.57; accuracy: *t*(15) = 2.13; *p* = 0.024, one-tailed; Cohen’s *d* = 0.53), while 7-Hz tACS significantly reduced K-values (and accuracy) relative to sham (*t*(15) = −1.78; *p* = 0.047, one-tailed; Cohen’s *d* = 0.44; *t*(15) = −1.83; accuracy: *p* = 0.046, one-tailed; Cohen’s *d* = 0.46) (Fig 1C leftmost graphs for mean and individual data and S1 Fig).

As expected, analysis of the right hemifield trials showed no significant effect of Condition (K: F(1,15) = 0.12; η^2^ = 0.008; *p* = 0.73; accuracy: F(1,15) = 0.002; *p* = 0.97; η^2^ = 0.0001), as well as no significant effect of Load or interactions (all *p* > 0.19) (Fig 1C, rightmost graphs and S1 Fig). Finally, the same analysis performed on the Control Montage showed no main effects or interactions reaching significance (all *p* > 0.23) (Fig 1D, S1A and S1B Fig).

**Fig 2:**
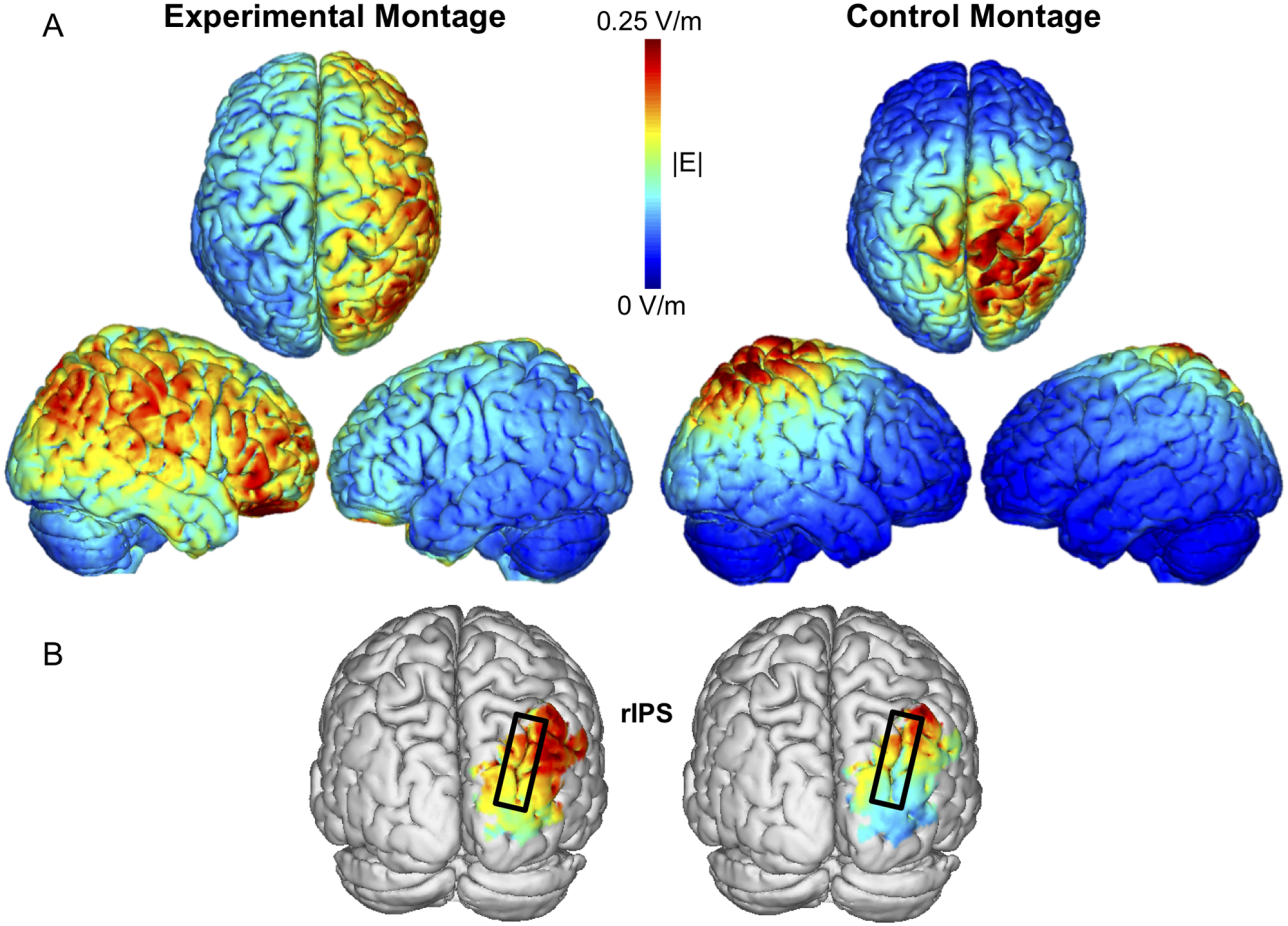
**(A) Electric field distribution calculation** (NIC 2.0 Software: http://www.neuroelectrics.com/products/software/nic2/) for Experimental (left) and Control (right) electrode montages. The Experimental Montage (P4-supraorbital) shows (i) a more right-lateralised field distribution with (ii) maximum current over more posterior parietal areas relative to the control montage (P4-Cz). The Control Montage shows (i) some left-lateralised field distribution with (ii) maximum current over right superior parietal areas, thus more anterior to P4. These differences in electric field distribution might be responsible for the significantly different impact obtained across the two montages and may possibly explain the null effects obtained using the Control Montage. **(B) Masked display with highlights of right IPS electric field distribution calculation** (NIC 2.0 Software: http://www.neuroelectrics.com/products/software/nic2/), relative to the Experimental (left) and Control Montages (right). Here, it can be more closely appreciated how the Experimental Montage induced a maximum current over IPS, relative to the Control Montage. IPS, intraparietal sulcus; NIC, Neuroelectrics Instrument Controller; rIPS, right intraparietal sulcus.

In order to clarify the contribution of the electrode configuration on the observed effects, we calculated electric field distribution for both montages based on a realistic head model [21]. Results of this analysis suggest that stimulation in the Control Montage (P4-Cz) led to more superior parietal stimulation and more left parietal stimulation, relative to our Experimental Montage (P4-supraorbital), in which participants received stronger stimulation exactly at around the right IPS [18] that then spread throughout the right hemisphere but was confined within it (Fig 2).

## Discussion

In this study, we show for the first time that by applying tACS over the parietal cortex at a slow theta frequency (4 Hz), we were able to boost visuospatial WM capacity. On the other hand, stimulating at a faster theta frequency (7 Hz) reduced visuospatial WM capacity. Modulation of WM capacity was selective for stimuli presented in the hemifield contralateral to the stimulated hemisphere, in line with previous reports [3,7,8,9] showing a lateralised parietal activation during stimulus retention, which correlates in turn with individual WM capacity. Moreover, these effects were observed only when the right parietal electrode was paired with the right supraorbital return electrode (Experimental Montage) but not with the Cz return electrode (Control Montage). Importantly, these results lend behavioural support to the seminal work by Lisman and Idiart [1], suggesting that a slower theta frequency would code for a higher number of gamma cycles, leading to enhanced WM capacity, whereas a faster theta frequency codes for a smaller number of gamma cycles, leading to reduced WM capacity.

### On the nature of tACS effects

The effects we obtained were in line with the expected empirical results. Theoretically, the difference between cycle length of 4 Hz (250 ms) and 7 Hz (143 ms) would be around 107 ms and would therefore allow for at least two additional gamma cycles to be nested into a theta cycle. Based on this evaluation, a difference of 2 items between stimulation conditions could be expected when stimulating at 4 Hz relative to 7 Hz. However, we note that the modulation of the K-values obtained here at 4 Hz and 7 Hz does not perfectly match the impact that would be expected from theory (see Fig 1A). Specifically, we found a total difference across conditions of about 0.8 items, which in terms of accuracy corresponds to about a 7% difference between 4 Hz and 7 Hz stimulation. This difference between the potential maximum impact of stimulation and the observed effect can be explained by the fact that tACS delivers very weak currents that might only partially drive the endogenous ongoing oscillatory activity. There is also recent evidence showing how the impact of tACS on oscillatory activity is strictly dependent on the state of the brain during stimulation [22]. This implies that intervening factors can also induce subtle modulation of tACS effects, rendering the stimulation less effective. For example, in our experimental manipulation, tACS was not delivered in phase with the beginning of the 900-ms retention period shown to be more strictly related to theta synchronization [2]. This might account for some trial-by-trial variability in the impact of theta tACS for maximising cross-frequency coupling in the relevant theta phase (i.e., stimulation may have been more effective in those trials in which tACS was in phase with the onset of the retention interval than in other trials that were less in phase or even in counterphase with the onset of the retention period). Moreover, one would only expect a full 2-item difference if each participant responded perfectly. Yet, individual differences might significantly interact with the way the method optimally works, and therefore one would also expect overall smaller mean values. However, despite such potential intervening factors, tACS still significantly impacted current results in the predicted direction.

### WM capacity and theta oscillatory peak: A stable, trait-like phenomenon, with functionally significant variations around the mean

WM capacity shows variability both between and within participants. Between-participants variability in WM capacity has been documented. For example, it has been shown that WM variance can be explained by the theta:gamma frequency ratio [23], and even within participants, trial-by-trial variability can be observed [24], with the same items being sometimes retained and sometimes not. We speculate that this variability might be reflected in slight variations in oscillatory activity related to this function. Indeed, recent reports support the functional relevance of the between and within trial-by-trial variability in frequency speed in different domains, from visual processing [11,25] to multisensory binding [12] and pain perception [26], thus rather discarding the notion of this being sheer noise in oscillatory fluctuations. Although not directly related to our experimental paradigm or specific oscillatory frequency, these findings provide a more general framework in support and physiological backup to the behavioural results we present here, suggesting that tACS can effectively shift individual trait-like behaviour associated with oscillatory activity in desired directions. In the context of WM capacity, the model of Lisman and Idiart [1] very well matches the theoretical framework of inter- and intraindividual oscillatory variability determining WM capacity, and we have indeed tested this in the current study. Therefore, while WM capacity is a trait-like ability, possibly centred around a person’s individual theta frequency peak, this trait-like dimension may be prone to slight but functionally significant fluctuations around the mean, which could be best explained by the trial-by-trial variability in the theta frequency peak, accounting for the trial-by-trial ability to correctly encode information in WM.

### Interindividual variability in theta frequency may impact the way tACS modulates WM capacity

Individual theta frequency may vary across participants in the full range between 3 and 8 Hz, thus even beyond the range of 4–7 Hz that we have considered. This might in principle explain why not all of our participants showed a consistent effect of enhanced or reduced WM capacity relative to their sham condition. According to our hypothesis, one would expect that 4-Hz tACS would always improve performance relative to sham and 7-Hz tACS would always reduce performance relative to sham. While at first sight, this might seem to be the case, this conclusion would be based on the misleading assumption that, at the individual level, sham stimulation would necessarily sit in the middle as if it corresponded to a 5.5-Hz stimulation. However, as per definition, individual theta frequency may vary within an even wider range than the one we have defined here, conventionally and conveniently, but arbitrarily, of 4 and 7 Hz. Indeed, while literature generally refers to theta as an oscillatory activity in a range between 4 and 7 Hz, it has been reported that both slow theta 3-Hz oscillations and fast theta 8-Hz oscillations can be associated with memory performance [27]. Therefore, 4- and 7-Hz stimulations do not necessarily sit on the lower and upper boundaries of the individual theta frequency. In turn, this may explain in principle why some participants do not show positive Sham-corrected values for 4-Hz stimulation with negative differential scores for 7-Hz stimulation at the same time, or being close to 0 in other cases. Crucially, this perspective would also explain why, in all cases, 4-Hz relative to 7-Hz stimulation always resulted in a better WM performance.

At an additional level of analysis, the shape of the individual theta peaks may also vary quite substantially from sharp to broad across individuals, which might in turn determine the net effect size of our data. Indeed, such characteristics may well interact in the way participants respond to the tACS interventions, such that participants with broadband theta might be more susceptible to tACS interference than those with sharp peaks. While we cannot provide a more detailed and conclusive demonstration of this mechanism here, these are all relevant points that future research needs to address. However, the current study already provides a fundamental step forward in the understanding of the mechanisms underlying spatial WM processing, showing the behavioural impact of tACS modulation of WM capacity closely following a theoretical model of WM capacity on the one hand and interventional impact of tACS on the other hand.

### The theta–gamma phase coupling hypothesis

It might be argued that a way to alternatively demonstrate the impact of tACS on WM capacity would instead be to modulate gamma frequency. So, in principle, stimulating at faster gamma frequency might enhance WM capacity, while stimulating at slower gamma frequency would instead reduce WM capacity. However, the theta–gamma coupling framework of WM capacity does not assume any changes in gamma frequency and, indeed, there is clear evidence against this. For instance, Axmacher and colleagues [2] showed that increasing WM load leads to a reduction in theta frequency, whereas gamma frequency is not significantly slowed down. Also, the theoretical framework assumes that single memory item representations would be reflected by activity in local gamma networks oscillating at exactly this gamma frequency. The single representations themselves would not change, therefore gamma frequency should not either. In support of this notion, there is evidence that locally generated gamma would not even change frequency if the network size were increased [28].

### Electric field modelling reconstruction: A role for frontal versus parietal stimulation?

Based on the electric field modelling reconstruction, the Experimental Montage shows its maxima over IPS, exactly underneath the stimulation electrode, as one would expect. The control condition shows instead a maximum more anterior, off the stimulation electrode, with a clearly less lateralised distribution of the electric field, which together could explain why, within our paradigm, there was no significant modulation of WM capacity in the Control Montage.

When looking at the Experimental Montage electric field distribution, this is clearly lateralised with maxima over the IPS and spread more widely through the right hemisphere, including frontal areas. According to this picture, it could be argued that the actual significant impact of the Experimental Montage might be due (i) to a more effective tACS of the right prefrontal cortex via the supraorbital electrode of the Experimental Montage or (ii) to a more effective activation of the frontoparietal network instead and not the P4 stimulation site per se, common to both montages, or even (iii) subcortical activations. Although we cannot completely rule out these alternative hypotheses, we argue that these are very unlikely explanations of current results. First, the right supraorbital electrode is not the optimal position for modulating frontal theta oscillatory activity (see previous tACS work targeting the frontoparietal network, with the frontal sensor sitting more posteriorly, e.g., over FCz or laterally over F3 and F4 [6,29,30]). Moreover, tACS montages testing the relative impact of frontal and parietal areas have shown a selective modulation of WM by parietal but not frontal stimulation [29,30]. Therefore, our Experimental Montage has likely not desynchronised frontal and parietal areas that were actively involved in the WM task but instead optimised a lateralised stimulation of one of the crucial nodes (right parietal area in this case) leading to current lateralised effects. Furthermore, if any of the effects observed could be ascribed to activation of frontal or even subcortical activations, it is unlikely to carry lateralised effects, which should instead be driven more specifically by parietal activations. Importantly, the choice of the parietal area was strongly inspired by relevant empirical work [3,7,8,9]. These studies showed that the spatial component is a relevant one in our experimental design that actively calls into play parietal rather than frontal activations. Indeed, both theta oscillations [3] and event related potential (ERP) components associated with spatial WM capacity [7,8,9], in the very same paradigm we have used here, are systematically localised contralateral to the items to be remembered and are crucially over posterior areas, the very same we have stimulated in our study. Instead, no such modulation of theta oscillations [3] or ERP [7,8,9] over prefrontal areas has been found whatsoever. Indeed, we are considering here a specific visuospatial cued memory component that strongly relies on the visuospatial components, for which parietal areas are primarily involved. It is very unlikely that such lateralised effects observed here and replicated numerous times (see, e.g., [31,32]) in different visuospatial WM experimental paradigms (see, e.g., [33,34]) may be driven by frontal activations. Therefore, based on this evidence, we explicitly expected the effects to be mainly driven by parietal stimulation and to be lateralised.

### Cortical versus retinal effects

One could argue that the effect we observed could be the result of retinal activation [16,35] due to supraorbital electrode stimulation. Several arguments, however, discount this alternative hypothesis. First of all, none of the participants saw any phosphenes during the experiment. Indeed, reports of retinal phosphene for stimulation frequency within the theta band are very rare (see [36]). Those few participants who saw phosphenes at the beginning (*n* = 3) were stimulated during the experiment at a tACS intensity not inducing any phosphene sensation. Yet, if one has to consider the potential impact of some residual retinal phosphenes perception over the WM capacity using the current paradigm, a different pattern of results would be expected than the one we currently observe. Specifically, we would expect any effect to be essentially ipsilateral to the stimulation site rather than contralateral, as observed here, instead. While we do observe a lateralised effect of stimulation on WM capacity, no main effect of hemifield could be detected, but only an interaction between hemifield and the specific frequency of stimulation. Such effects were observed for the contralateral rather than ipsilateral hemifield to the stimulated hemisphere and retina and are thus unlikely to be explained by retinal activation.

Finally, if phosphenes were not perceived, it might still be argued that a subthreshold impact of tACS on retinal activity might be induced, which may in turn indirectly induce cortical entrainment in corresponding visual areas. If this were to be the case, we would still argue that the activation of the ipsilateral retina would project onto both hemispheres, thus inducing a bilateral entrainment, which is not compatible with the lateralised effect we observed in the current study. Therefore, we have good reasons to believe that any of the effects we observed were genuinely cerebral in nature.

### Conclusions

To conclude, we found that, depending on electrode configuration, 4-Hz tACS enhanced visuospatial WM capacity, while 7-Hz tACS reduced visuospatial WM capacity compared to sham stimulation. As a result of the hemifield specificity effect, each participant served as their own internal control, depending on the hemifield being tested relative to the hemisphere being stimulated, with the effects being found for items presented to the hemifield contralateral to the stimulation site only. These results are also supported by recent reports in monkeys [37] performing a similar change detection task and showing an independence of the two hemispheres for visual WM function.

The findings of this study are in line with the theta–gamma phase coupling theory of WM capacity [1]. While direct electrophysiological evidence supporting our conclusions is still lacking due to the technical challenge of combining online tACS and EEG (see [6,10]), here we provide relevant behavioural evidence that slow theta tACS enhances visuospatial WM capacity and fast theta tACS reduces visuospatial WM capacity, in line with the idea that slow and fast theta tACS might induce slower and faster theta oscillations, respectively (see [10,11,12,13]). These findings may provide the basis for potential therapeutic interventions aimed at enhancing poor memory capacity in the ageing population and to ameliorate memory-related neuropathologies in clinical settings.

## Materials and methods

### Ethics statement

The study has been approved by the University of Essex Ethics Committee (VR1301) and conducted according to the principles expressed in the Declaration of Helsinki. Written informed consent has been obtained from the participants before taking part in the study.

### Participants

Based on power analysis (conducted on [6], actual effect size, f = 0.47), with a conservative estimated effect size f = 0.25, alpha = 0.05, and 80% power, a total sample size of 28 participants (14 per group) is suggested. Therefore, in a between-subjects design, we assigned 16 participants (7 female) to the Experimental Montage and 16 (9 female) to the Control Montage. All participants were adults (18 years or older) with mean ages of 28.3 years (±7.6) and 22.8 years (±5.2), respectively. Participants completed a safety screening questionnaire before taking part in each session of the study.

### Design

A repeated measures design was used for stimulation conditions and memory load. There were two active stimulation conditions (4- and 7-Hz tACS) and one control condition (sham). These stimulation conditions took place during three separate sessions that occurred on separate days, and the order was counterbalanced across participants. At least 24 hours passed between different sessions. A single-blind design was used, with participants unaware that different stimulation protocols were being used for each session (and unaware that one session consisted of sham stimulation).

### Stimulation protocol

#### Experimental Montage

Two 5 × 7 cm electrodes were placed over the scalp using saline-soaked sponges. Prior to electrode placement, skin was prepped with alcohol wipes to improve conductivity. One electrode was placed over the right parietal cortex (corresponding to P4, according to the 10–20 system) and the other was placed on the forehead above the right eyebrow (supraorbital). Maximum stimulation intensity was initially set to 1,500 μA peak-to-peak amplitude and was delivered using a Magstim DC-Stimulator Plus device.

During the first session, stimulation at 1,500 μA was tested to see if retinal phosphenes occurred. If none occurred, this level of stimulation was used. If retinal phosphenes occurred at this level, the individual threshold for retinal phosphenes was determined for that participant. This was done by starting at 1,000 μA and increasing in increments of 25 μA or decreasing in increments of 100 μA and then back up in increments of 25 μA until the highest level that did not produce retinal phosphenes was found. The average level of stimulation was 1,238 μA (±298 μA). For the second and third sessions, the voltage determined in the first session was used. In three cases, retinal phosphenes were reported at this level during the second or third session and the voltage was therefore reduced for the remaining sessions for those three participants (using the method outlined above to determine the appropriate level). In these cases, this resulted in differences of −50 μA, −650 μA, and −400 μA for the 7-Hz session relative to the 4-Hz session. No other participants reported retinal phosphenes at the threshold determined during the first session.

All conditions consisted of a ramping up stage at the beginning of the stimulation. For the 4-Hz condition, this lasted for 120 cycles; for the 7-Hz condition, this lasted for 210 cycles; and for the sham condition, this lasted for 165 cycles. For the 4- and 7-Hz conditions, this was followed by stimulation at the predetermined threshold or at 1,500 μA; for the sham condition, the ramping up period was followed by stimulation for 165 cycles and then a fade-out period that lasted 165 cycles. The brief stimulation period in the sham condition was carried out to mimic any sensations that may have been felt during the active stimulation conditions. This also allowed the retinal phosphene threshold to be determined when the first session contained sham stimulation. During the ramping-up, stimulation, and fade-out periods in the sham condition, the frequency of stimulation was at 5.5 Hz. This frequency was chosen as a neutral frequency relative to the two active conditions. During the sham session, participants performed the task only after the fade-out period. Throughout all stimulation sessions, impedance levels were kept below 5 kΩ.

#### Control Montage

Two 5 × 7 cm electrodes were placed over the scalp using saline-soaked sponges. Prior to electrode placement, skin was prepped with alcohol wipes to improve conductivity. One electrode was placed over the right parietal cortex (corresponding to P4, according to the 10–20 system) and the other was placed on the vertex (corresponding to Cz, according to the 10–20 system). Stimulation was delivered using a Magstim DC-Stimulator Plus device. Stimulation was delivered at 1,500 μA. None of the participants reported retinal phosphenes at this level during any session.

All conditions consisted of a ramping up stage at the beginning of the stimulation, and the procedure for this was the same as with the Experimental Montage. As with the Experimental Montage, the frequency of stimulation during the ramping up and fade-out periods in the sham condition was 5.5 Hz and the total duration of stimulation was 495 cycles, inclusive of the ramping up and down periods. Throughout all stimulation sessions, impedance levels were kept below 5 kΩ.

### Materials

A variation of the visual delayed match to sample task based on Vogel and Machizawa [8] was used. Two arrays of coloured squares were situated on either side of a white fixation cross in the centre of a black screen. The number of squares in each array (the memory load) was 4, 5, or 6. The left and right arrays were always different from each other in any given trial; however, the number of squares in each trial was always the same for the left and right arrays.

#### Behavioural task

The task started with a fixation cross on the screen. Prior to presentation of the arrays, an arrow briefly appeared on the screen (200 ms) to indicate which of the two upcoming arrays (left or right) needed to be memorised. The two arrays (one on the left and one on the right of the fixation cross) then appeared on the screen for 100 ms. This was followed by a retention interval of 900 ms, during which there was a fixation cross on the screen. After the retention interval, two arrays appeared on the screen for 2,000 ms (again, there was one array on either side of a fixation cross). Participants had to indicate whether this array on the previously indicated side was the same as or different than the array on that side presented previously during that trial. This was done by pressing either the left or right button of the mouse (left to indicate that the arrays were the same and right to indicate that they were different). The order of trials within each block was randomised by the computer program.

There were 6 blocks of 20 trials presented during each stimulation session. Each block contained trials of one load only (4, 5, or 6). For each session, the blocks were chosen at random from a set of 9 blocks (3 for each load) with the following restrictions: exactly 2 blocks of each load were to be used and no block was to be repeated during any given session. These 6 blocks were presented in a randomised order. Each block contained 5 left-mismatched, 5 left-matched, 5 right-mismatched, and 5 right-matched trials.

#### Experiment procedure

After the setup of the electrodes, participants were presented with written instructions. Participants were instructed to use only one hand to make their responses and were given the opportunity to clarify the instructions if needed. The lights were turned off and participants carried out a practice block of trials. This practice block used load 3 stimuli to prevent load-specific practice effects from affecting subsequent performance.

After the practice block had finished, additional saline solution was added to the sponges and stimulation was started. Participants were then instructed to start the first block when ready. After each experimental block, participants pressed the enter button to continue to the next block. Each block lasted approximately 2 minutes. Therefore, the total task time during stimulation lasted approximately 12 minutes. Participants were given monetary compensation or course credits for their participation after each of the three sessions and were debriefed after the final session and invited to ask any questions.

#### Data analysis

Participant responses were preprocessed before undergoing statistical analysis. A mixed repeated measures design was used with the between-factor Montage and the within-factors Load, Hemifield, and Condition (Stimulation Frequency).

Because there were three different load conditions (4, 5, and 6), a simple analysis of the correct response was not optimal, and instead, a K-value was implemented as a dependent variable (in addition to accuracy). K is a standardised measure [20] of WM capacity that takes set size into account (e.g., [3,7,8,9,20,34,38,39,40]) and is defined as K = S * (H − F), where H is the hit rate (i.e., the percentage of trials correctly recognised as the same or different), F is the false alarm rate (i.e., the percentage of trials erroneously judged as the same when they are different and vice versa), and S is the set size (i.e., the number of items presented in each trial, which could be either 4, 5, or 6 in the current task). K-values (and accuracy) were determined for each Montage, Condition, Load, and Hemifield combination.

The within-subjects design included two active (4- and 7-Hz) and one sham stimulation condition. The two active conditions were normalised to the sham condition (4 Hz minus sham and 7 Hz minus sham) for each montage, load, and hemifield (for completeness, non-sham-corrected data are reported in S1 Fig). A mixed repeated measure ANOVA was conducted with the between-subjects factor Montage (Experimental and Control Montage) and the within-subjects factors Condition (Sham-corrected 4 Hz, Sham-corrected 7 Hz), Load (4, 5, and 6), and Hemifield (left and right). When interactions reached significance, subsequent ANOVAs were performed separately for Montage and Hemifield, followed up by direct contrast paired *t* test between Sham-corrected 4 and 7 Hz, when appropriate. Sham-corrected 4 and 7 Hz were also submitted to a one-sample *t* test against 0 to test whether they significantly differed from sham. One-tailed *t* tests were used as we anticipated directionality of the effects for the 4- and 7-Hz stimulation relative to each other and to sham, based on the theoretical model and empirical evidence upon which the experimental design was conceived.

Neuroelectrics Instrument Controller (NIC) 2.0 software was used to calculate electric field distribution. The software is based on the approach developed and described by Miranda and coworkers [21]: In NIC 2.0, the electric field E at the interface between cerebrospinal fluid and grey matter was estimated in an MRI-based realistic head model [21]. This head model was built from a Colin 27 template obtained from BrainWeb [21]. In this model, different kinds of tissue were modelled as homogeneous and isotrophic media. Electrical conductivity was estimated with 0.33 S/m for the scalp and grey matter, 0.15 S/m for white matter, 0.008 S/m for the skull, and 1.79 S/m for cerebrospinal fluid. The sponge electrodes were estimated with a conductivity of 2 S/m [21]. At the interface between cerebrospinal fluid and grey matter, a finite element mesh was created using Comsol software. Based on Comsol software, Laplace’s equation for the electric potential was used. Then, the electric field at all nodes of the mesh was estimated by using the gradient of the electric potential [21]. In Fig 2, the magnitude of the electric field |E| is displayed separately for the Experimental Montage (P4-supraorbital) and the Control Montage (P4-Cz).

## Supporting information

S1 Fig Analysis performed on non-sham-corrected (A) K-values and (B) accuracy for the Experimental and Control Montages: Results (underlying data can be found at: https://osf.io/rm6qp/). Leftmost graphs depict mean K-values and accuracy obtained for trials presented on the left hemifield for each active (4- and 7-Hz) and sham condition, while rightmost graphs depict mean K-values and accuracy obtained for trials presented on the right hemifield for each active and sham condition. Significant differences between stimulation conditions were observed for the Experimental but not for the Control Montage and only for stimuli presented to the left hemifield, leftmost graph (i.e., contralateral to the stimulated parietal site). *p < 0.05; ****p < 0.0001. Error bars depict standard error of the mean. Unlike the sham-corrected analysis presented in the main text, this data analysis does not factor out the variability induced by including the control factor “sham” in the between-group design, resulting in weaker between-group effects. The mixed factorial ANOVA with the between-factors Montage (Experimental versus Control) and within-factor Condition (4 Hz, 7 Hz, and Sham) × Load (4, 5, and 6 items) × Hemifield (left and right) carried out on the K-values and accuracy showed an effect of Load (K: (F2,60) = 26.4; *p* < 0.00000001; η^2^ = 0.47; accuracy: F(2,60) = 82.848; *p* < 0.00000000001; η^2^ = 0.734), confirming that the task is generally more challenging for higher than lower loads. A Condition × Hemifield × Montage marginal interaction (K: (F2,60) = 2.72; *p* = 0.074; η^2^ = 0.083; accuracy: F(2,60) = 2.47; *p* = 0.093; η^2^ = 0.076) suggests that the two montages had a different impact on performance, depending on stimulation Condition and Hemifield. To further ascertain the nature of the impact of montage on frequency-specific effects, the same ANOVA was performed separately for each group. In the Experimental Montage, we confirmed an effect of Load (K: F(2,30) = 19.12; *p* < 0.000001; η^2^ = 0.56; accuracy: F(2,30) = 52.55; *p* < 0.00000001; η^2^ = 0.78. In addition, we found a main effect of Condition (K: F(2,30) = 3.29; *p* = 0.05; η^2^ = 0.18; accuracy: F(2,30) = 3.69; *p* = 0.036; η^2^ = 0.20) and a significant interaction Condition × Hemifield (K: F(2,30) = 3.70; *p* = 0.037; η^2^ = 0.198; accuracy: F(2,30) = 3.61; *p* = 0.039; η^2^ = 0.194), suggesting that the different stimulation conditions had a differential impact on left and right hemifields. These trials were analysed separately in two further repeated measures ANOVAs (Condition × Load). As expected, the analysis of the left hemifield trials showed again an effect of Load (K: F(2,30) = 5.49; *p* < 0.01; η^2^ = 0.36; accuracy: F(2,30) = 20.42; *p* < 0.00000001; η^2^ = 0.58) and a significant main effect of stimulation condition (K: F(2,20) = 8.57; *p* = 0.0011; η^2^ = 0.364; accuracy: F(2,30) = 9.94; *p* < 0.001; η^2^ = 0.40), while no significant interaction between Load and Condition was observed (K: F(4,60) = 0.60; *p* = 0.66; η^2^ = 0.038; accuracy: F(4,60) = 0.185; *p* = 0.94; η^2^ = 0.012). Subsequent *t* tests revealed 4-Hz stimulation to induce higher K-values and accuracy compared to sham condition (K: *t*(15) = 2.28; *p* = 0.019, one-tailed; Cohen’s *d* = 0.57; accuracy: *t*(15) = 2.13; *p* = 0.024; Cohen’s *d* = 0.53); 7-Hz stimulation to induce lower K-values and accuracy compared to sham condition (K: *t*(15) = 1.79; *p* = 0.047, one-tailed; Cohen’s *d* = 0.45; accuracy: *t*(15) = 1.83; *p* = 0.043, one-tailed; Cohen’s *d* = 0.46); and 4-Hz stimulation to induce higher K-value and accuracy compared to 7-Hz stimulation condition (K: *t*(15) = 5.11; *p* = 0.000065, one-tailed; Cohen’s *d* = 1.278; accuracy: *t*(15) = 6.75; *p* < 0.00001; Cohen’s *d* = 1.69) (leftmost graphs). Analysis of the right hemifield trials showed only a main effect of Load (K: F(2,30) = 12.41; *p* < 0.0001; η^2^ = 0.45; accuracy: F(2,30) = 37.22; *p* < 0.00000001; η^2^ = 0.71) but no other main effects or interactions (all *p* > 0.21) (rightmost graphs). The same analysis performed on the Control Montage showed only an effect of Load (K: F(2,30) = 9.39; *p* = 0.00068; η^2^ = 0.385; accuracy: F(2,30) = 33.40; *p* < 0.00000001; η^2^ = 0.69), with no other main effects or interactions reaching significance (all *p* > 20).
(TIFF)

S1 Table (A) K-values and (B) accuracy values (%) for each montage (Experimental and Control Montages), stimulation condition (4 Hz, 7 Hz, and Sham), Hemifield (left and right), and Load (4, 5, and 6 items) (underlying data can be found at https://osf.io/rm6qp). (XLSX)

## Abbreviations

EEG: electroencephalography
ERP: event related potentials
IPS: intraparietal sulcus
MEG: magnetoencephalography
NIC: Neuroelectrics Instrument Controller
tACS: transcranial alternating current stimulation
TMS: transcranial magnetic stimulation
WM: working memory
