# Supplementary figures and images for: The speed of parietal theta frequency drives visuospatial working memory capacity
Source: PLoS Biol. 2018 Mar 14;16(3):e2005348. doi: 10.1371/journal.pbio.2005348 (PMC5868840; doi:10.1371/journal.pbio.2005348)

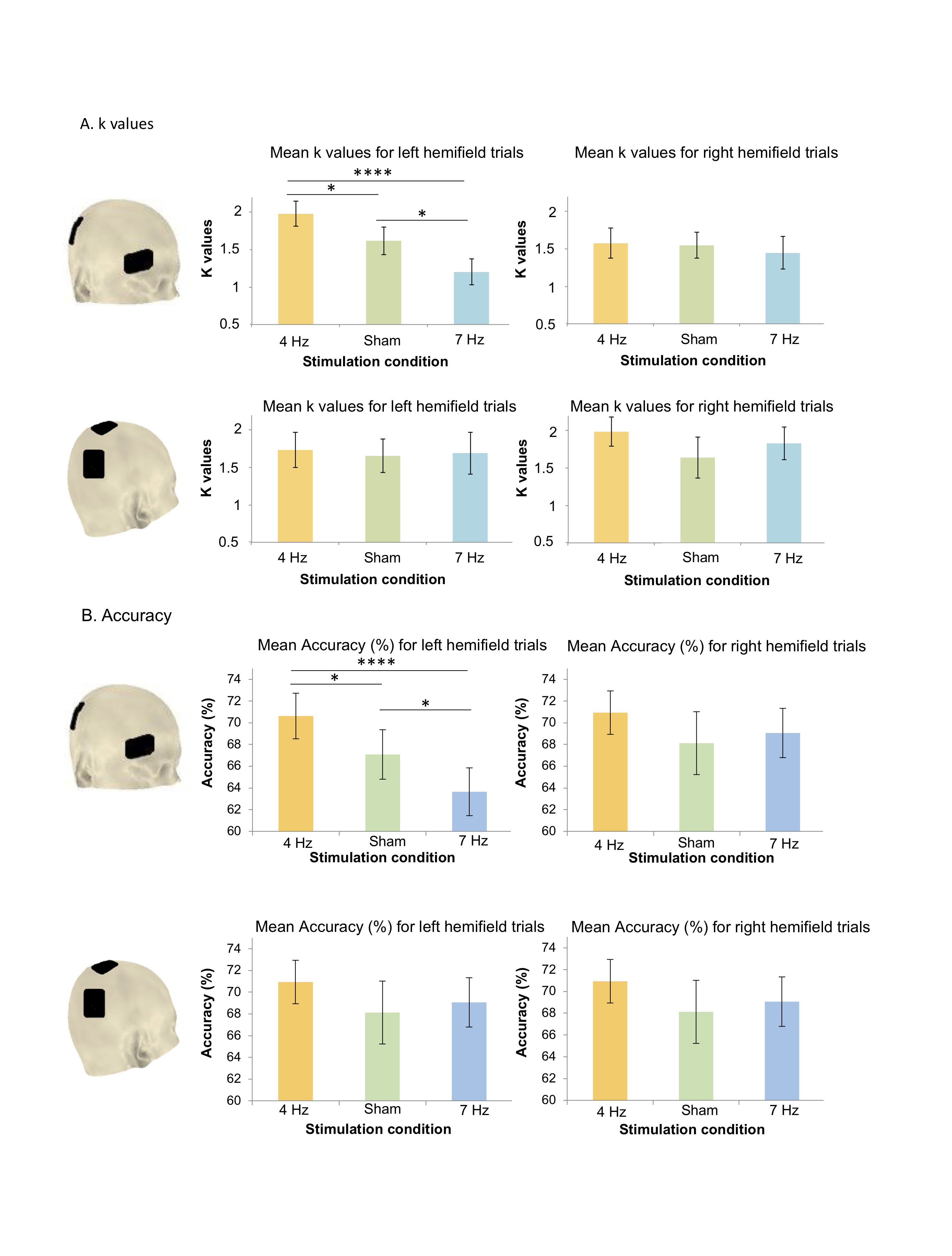

Supplement: S1 Fig — Leftmost graphs depict mean K-values and accuracy obtained for trials presented on the left hemifield for each active (4- and 7-Hz) and sham condition, while rightmost graphs depict mean K-values and accuracy obtained for trials presented on the right hemifield for each active and sham condition. Significant differences between stimulation conditions were observed for the Experimental but not for the Control Montage and only for stimuli presented to the left hemifield, leftmost graph (i.e., contralateral to the stimulated parietal site). *p < 0.05; ****p < 0.0001. Error bars depict standard error of the mean. Unlike the sham-corrected analysis presented in the main text, this data analysis does not factor out the variability induced by including the control factor “sham” in the between-group design, resulting in weaker between-group effects. The mixed factorial ANOVA with the between-factors Montage (Experimental versus Control) and within-factor Condition (4 Hz, 7 Hz, and Sham) × Load (4, 5, and 6 items) × Hemifield (left and right) carried out on the K-values and accuracy showed an effect of Load (K: (F2,60) = 26.4; p < 0.00000001; η2 = 0.47; accuracy: F(2,60) = 82.848; p < 0.00000000001; η2 = 0.734), confirming that the task is generally more challenging for higher than lower loads. A Condition × Hemifield × Montage marginal interaction (K: (F2,60) = 2.72; p = 0.074; η2 = 0.083; accuracy: F(2,60) = 2.47; p = 0.093; η2 = 0.076) suggests that the two montages had a different impact on performance, depending on stimulation Condition and Hemifield. To further ascertain the nature of the impact of montage on frequency-specific effects, the same ANOVA was performed separately for each group. In the Experimental Montage, we confirmed an effect of Load (K: F(2,30) = 19.12; p < 0.000001; η2 = 0.56; accuracy: F(2,30) = 52.55; p < 0.00000001; η2 = 0.78. In addition, we found a main effect of Condition (K: F(2,30) = 3.29; p = 0.05; η2 = 0.18; accuracy: F(2,30) = 3.69; p = 0. [file pbio.2005348.s001.tiff]
